# Optimizing water relations, gas exchange parameters, biochemical attributes and yield of water-stressed maize plants through seed priming with iron oxide nanoparticles

**DOI:** 10.1186/s12870-024-05324-w

**Published:** 2024-06-29

**Authors:** Muhammad Waqas Mazhar, Muhammad Ishtiaq, Mehwish Maqbool, Khursheed Muzammil, Ali Mohieldin, Adam Dawria, Abdelrhman Ahmed Galaleldin Altijani, Ahmed Salih, Omar Yousof M. Ali, Ahmed Abdelgadir Mohamed Elzaki, Bhgah I. Yusuf Adam, Hamza Abdullah M. Adam

**Affiliations:** 1 Department of Botany, Mirpur University of Science and Technology, Mirpur, AJK 10250 Pakistan; 2 Department of Botany, Climate Change Research Centre, Herbarium and Biodiversity Conservation, Azad Jammu and Kashmir University of Bhimber (AJ&KUoB), Bhimber, 10040 AJK Pakistan; 3 Department of Public Health, College of Applied Medical Sciences, King Khalid University, KhamisMushait Campus, Abha, Saudi Arabia; 4 Department of Public Health, College of Health Sciences, Saudi Electronic University, Riyadh, Saudi Arabia; 5 Public Health Department, Faculty of Applied Medical Sciences, Al Baha University, Al Baha, Saudi Arabia; 6 Health Education and Promotion Department, College of Public Health and Tropical Medicine, Jazan University, Jazan, Kingdom of Saudi Arabia; 7 Department of Epidemiology, College of Public Health & Tropical Medicine, Jazan University, Jazan, Saudi Arabia

**Keywords:** Iron oxide nanoparticles, Nano-priming, Maize plants, Water use efficiency, Drought stress

## Abstract

Drought poses significant risks to maize cultivation by impairing plant growth, water uptake and yield; nano priming offers a promising avenue to mitigate these effects by enhancing plant water relations, stress tolerance and overall productivity. In the current experiment, we tested a hypothesis that seed priming with iron oxide nanoparticles (n-Fe_2_O_3_) can improve maize performance under water stress by improving its growth, water relations, yield and biochemical attributes. The experiment was conducted on a one main plot bisected into two subplots corresponding to the water and drought environments. Within each subplot, maize plants were raised from n-Fe_2_O_3_ primed seeds corresponding to 0 mg. L^− 1^ (as control treatment), 25, 50, 75, and 100 mg. L^− 1^ (as trial treatments). Seed priming with n-Fe_2_O_3_ at a concentration of 75 mg. L^− 1^ improved the leaf relative water content, water potential, photosynthetic water use efficiency, and leaf intrinsic water use efficiency of maize plants by 13%, 44%, 64% and 17%, respectively compared to control under drought stress. The same treatments improved plant biochemical attributes such as total chlorophyll content, total flavonoids and ascorbic acid by 37%, 22%, and 36%, respectively. Seed priming with n-Fe_2_O_3_ accelerated the functioning of antioxidant enzymes such as SOD and POD and depressed the levels of leaf malondialdehyde and hydrogen peroxide significantly. Seed priming with n-Fe_2_O_3_ at a concentration of 75 mg. L^− 1^ improved cob length, number of kernel rows per cob, and 100 kernel weight by 59%, 27% and 33%, respectively, under drought stress. Seed priming with n-Fe_2_O_3_ can be used to increase maize production under limited water scenarios.

**Supplementary Information:**

The online version contains supplementary material available at 10.1186/s12870-024-05324-w.

## Introduction

Currently, agriculture is adjusting to new thermal regimes that have the potential to disrupt crop growth phases and the soil ecologies that sustain them, with particular repercussions for the spread of crop disease and frequent droughts [1]. Adjustments to rain-fed and irrigated output are being required as a result of fundamental changes to the water cycle, particularly in the patterns of rainfall and times of drought [2]. The land and water resourcesare being polluted and depleted continuously. There is need to improve crop water use efficiency and yield for global food security under changing climates. Furthermore, the over doses of fertilizers to croplands is bringing risk to aquatic lives through eutrophication and there is escalating risk of nutrient pollution as well [3]. There is need to adopt measures to increase crop yield with bio-rational and eco-friendly practices such as seed priming [4].

Nanotechnology involves manipulation of nanomaterials for human welfare. These nanomaterials are being used in agriculture, medicine and service industries worldwide with promising results [5]. The use of nanomaterials in agriculture aims to minimize the distribution of chemicals, decrease nutrient losses during fertilization and enhance agricultural productivity by improving pest and nutrient management strategies. With revolutionary nano agrochemicals for the control of quick disease diagnosis, boosting plant nutrient absorption and other uses, nanotechnology has the potential to advance the agricultural and food industries [6].

Seed priming induces physiological changes to the seed enabling better seed germination and seed vigour [7, 8]. Now a day, nanopriming is emerging as a fascinating area of exploitation in agriculture sector. Nano priming leads to formation of hydroxyl radicals which cause loosening of seed coats and helps in mobilization of starch contents [9]. Seed priming with nanomaterials induces expression of aquaporin genes and optimizes endogenous reactive oxygen species (ROS) levels leading to better seed germination and plant emergence. The optimum ROS levels are necessary to activate synthesis of secondary metabolites and stress tolerance mechanism [10].

*Zea mays* L., also known as corn or maize, is a significant annual grain crop in the world [11]. In many regions of the world, it is regarded as a staple food. After rice and wheat, it is the third most important crop in the world [12]. With an annual production of 3.5 million metric tons, maize is grown over more than one million hectares in Pakistan. The growth and production of maize is decreasing in Pakistan due to climate change mediated frequent droughts. There is need to increase the yield and production through climate smart agricultural practices [13, 14].

Iron is a crucial micronutrient for the growth of plants [15]. Due to its involvement in the production of chlorophyll and the defence of chloroplasts, it plays a crucial part. The distribution of plant species in natural environments is governed by the availability of iron, which also affects crop productivity and nutritional value [16]. Several studies have reported the use of n-Fe_2_O_3_in inducing stress mitigation mechanism in plants and improving yield profile. Maswada et al., [17] highlighted the positive outcomes of using n-Fe_2_O_3_ as nano seed priming agents and shown that doing so boosts the water content of the leaves and the biomass output of the sorghum plant. The positive effects of using n-Fe_2_O_3_ on *Capsicum anum* L. were reported by Kumar et al., [18] in their study.

There is limited documentation on the seed priming use of n-Fe_2_O_3_, particularly in maize, despite its importance as staple crop. In the light of literature presented we hypothesize that seed priming with n-Fe_2_O_3_ raises crop performance in terms of yield, biochemical attributes, osmolytes and biomass production optimizing water use efficiency in the context of climate change mediated land and water resource scarcity. This study aims to optimize the performance of maize plants under water stress by investigating the effects of seed priming with n-Fe_2_O_3_ on various physiological and biochemical parameters. Specifically, it seeks to evaluate the impact of this priming on water relations, including water use efficiency, as well as gas exchange parameters such as stomatal conductance and transpiration rates. Additionally, the study will assess biochemical responses such as chlorophyll content, stress indicators levels, and antioxidant enzyme activity, alongside analysis of pigments and osmolytes. The research will also measure yield components, including kernel count and weights and compare the effectiveness of nanoparticle priming against controls. By exploring the underlying physiological and molecular mechanisms, the study aims to develop practical recommendations for farmers, enhancing the resilience and productivity of maize in water-limited environments and promoting sustainable agricultural practices.

## Materials and methods

### Experimental setup and treatments

The experiment was carried out in natural climatic settings from March to June 2023 (average day and night temperatures were 39.2 °C and 23.5 °C, respectively). Day length was between 11 and 12 h, and the relative humidity ranged from 31.6 to 65.8% [19]. Split plots were used to arrange the entire experimental area [20]. Two major plots were created in the experiment’s allotted space, one for each irrigation scheme. Then, five subplots were created for each main narrative, one for each distinct therapy. The subplots included three equal-sized rows that were the replicates, separated by 75 cm between each row. The soil was properly prepared by ploughing when it reached field capacity and by applying the recommended amounts of N (160 kg/ha), P (80 kg/ha), and K (50 kg/ha).

Seeds of maize cv. pearl were purchased from Pakistan’s National Agricultural Research Institute (NARC) in Islamabad. Iron oxide or magnetite nanoparticles (n-Fe_2_O_3_) were bought from Alpha Genomics Plot 4 C, Main PWD Rd, Islamabad, Punjab Pakistan [4]. Particle sizes in the 10–40 nm range, a density of 5.2 kg/L, and a purity percentage of 97.6% were all disclosed by the suppliers. Different concentrations of n-Fe_2_O_3_ were generated for the seed priming treatment, including a control treatment of 0 mg. L^− 1^and treatments of 25,50, 75, and 100 mg. L^− 1^ (Table S1). For preparation of each, 25, 50, 75, and 100 mg. L^− 1^ treatment solution 25, 50, 75, and 100 mg of n-Fe_2_O_3_ were dissolved in 1 L of distilled water following Mazhar et al., [4]. The mixtures were subjected to 30-minute ultra-sonication to create homogenous dispersions, and the desired n-Fe_2_O_3_concentrations were then raised. The control seedlings were primed with continuous aeration treatment for 24 h under dark conditions while the remaining seeds were immersed in their respective concentration range [4]. The soil variables of the experimental plots are shown in Table 1.

**Table 1:** Studied soil variables of the experimental area

Parameter	Value
Soil Type	Loamy
Electrical conductivity	1.31 dS.m^− 1^
Total suspended solids	11.10 meq.L^− 1^
CO_3_^2−^	0.87 meq.L^− 1^
HCO_3_^1−^	5.84 meq.L^− 1^
Cl^− 1^	4.84 meq.L^− 1^
Na^+^	5.24 meq.L^− 1^
Ca^2+^	4.88 meq.L^− 1^
Sodium absorption ratio	2.84 meq.L^− 1^

In furrows, the seeds were manually sown Thinning was done eight days after seedling emergence tomaintain a plant distance of 30 cm. All of the plots were irrigated following the thinning.

Water deficit stress therapy was initiated by managing the irrigation timings after the seedlings had emerged for 15 days. The experiment, conducted over 18 weeks from March to June 2023, focused on the life cycle of maize plants, which encompasses four primary stages: Germination and Seedling (4 weeks), Vegetative Growth (5 weeks), Tasseling and Silking (4 weeks), and Grain Filling or Maturing (5 weeks).

Subplots labelled “well-irrigated” received one irrigation during the first stage and three irrigations during the subsequent three stages. On the other hand, maize plants labelled as “stressed” received one irrigation during the first stage, followed by one irrigation during each of the remaining three stages. This approach followed a deficit irrigation method, as outlined by Shehzad et al. [20].

The total amount of water for the ten irrigations equated to 1000 mm, while stressed subplot plants received 400 mm of water across four irrigations. The irrigation water had a pH of 7, an electrical conductivity of 0.89 dS m^− 1^, and a residual sodium carbonate of 1.83 meq L^− 1^. To maintain drought pressure, temporary rain shelters were erected to prevent rainfall.

Three replicates from each treatment were chosen to record various parameters throughout the experiment. Maize plant sampling for biochemical parameters took place after 14 weeks post-sowing, while growth and yield parameters were assessed at crop maturity on June 30, 2023 (18 weeks post-sowing).

### Plant water relations and gas exchange parameters

For the measurement of the leaf water potential (Ψ_w_), a completely grown third leaf from the top was removed and employed in a pressure chamber [20]. The same leaf was frozen at 20 °C for 8 days after the water potential was measured. Following the thawing of the frozen leaf, the cell sap was taken out. 10 uL of the cell sap was used to measure leaf osmotic potential (Ψs) using an osmometer. The turgor potential was observed by the following formula:

1 $${\rm{\Psi P = \Psi w - \Psi s}}$$

The leaf fresh weights from each treatment were recorded. Subsequently, the leaves were placed in distilled water for four hours. Leaves were then blotted for surface water and were weighed for turgid weights. Dry weights of the leaves were evaluated after heating them in an oven for 48 h at 70 °C. Leaf relative water content was studied using the following equation:

2 $$\begin{array}{l}{\rm{Leaf}}\,{\rm{relative}}\,{\rm{water}}\,{\rm{content}}\,{\rm{\% = }}\left[ {\left( {{\rm{Leaf}}\,{\rm{fresh}}\,{\rm{Weight - Leaf}}\,{\rm{dry}}\,{\rm{weight}}} \right)} \right.\\\left. {{\rm{/}}\left( {{\rm{Leaf}}\,{\rm{turgid}}\,{\rm{Weight - Leaf}}\,{\rm{dry}}\,{\rm{weight}}} \right)} \right]{\rm{ \times 100}}\end{array}$$

Gas exchange parameters including stomatal conductance (gs), net CO_2_ assimilation (A), and transpiration rate (E) were evaluated using a portable infrared gas analyser (IRGA) LCA4 ADC (Analytical Development Company, Hoddesdon, England). Calculations for these parameters were performed using a fully developed leaf located in the upper third of the plant. The estimation of the gas exchange parameters was conducted under an average light intensity ranging from 4.68 kWh/m^2^/d to 5.54 kWh/m^2^/d. To assess leaf water use efficiency, the ratio of net CO_2_ assimilation to transpiration rate (A/E) was determined. Additionally, leaf intrinsic water use efficiency was determined by dividing the net CO_2_ assimilation value by stomatal conductance (A/gs) [20].

### Analysis of pigments

Observation on maize’s chlorophyll concentration was appraised using the Lichtenthaler and Wellburn [21] method. Briefly, 0.05 g of fresh maize leaf samples were dissolved in 10 mL of 80% acetone (v/v), and the optical densities of the extract for chlorophyll a and chlorophyll b were measured at 663 and 645 nm, respectively.

The 50 mg of fresh maize leaves were ground in 250 ul of acidic methyl (1% HCl, W/V) to estimate the anthocyanin concentration. The grinding was centrifuged at 14,000 rpm at room temperature for 5 min. With the use of a UV-Vis Spectrophotometer, absorbance was measured at 530 and 650 nm. In order to estimate the anthocyanin content, the formula below was utilized.

3 $${\rm{Q}}\,{\rm{Anthocyanin}}\,{\rm{ = }}\,\left( {{\rm{A}}\,{\rm{530 \times A}}\,{\rm{657}}} \right)\,{\rm{x}}\,{\rm{M1}}$$

Where M is the weight of the plant material used for extraction, A 530 nm, and 657 nm are the absorption at the stated wavelengths, and Q Anthocyanin is the corrected absorption value linearly associated with the number of anthocyanin [22].

### Total soluble sugars and flavonoids determination

Fresh maize leaf samples were frozen at 10 °C and crushed in 0.1 M monobasic phosphate buffer to measure total soluble sugar. The extracts underwent filtering and a 15 min, 3000 rpm cold centrifugation. The protocol of Dubois et al. was used to determine the total soluble sugars present in samples using the phenol sulphuric acid [23] Flavonoid contents were appraised following Karadeniz et al., [24].

### Malondialdehyde (MDA) and hydrogen peroxide (H_2_O_2_) contents

A 0.5 g sample of frozen maize leaves was ground into a fine powder. Each sample was then ground in 5 mL of a 10% trichloroacetic acid (TCA) solution. This substance was then centrifuged for 15 min at 7000 rpm. The supernatant was utilized to evaluate the MDA after centrifugation [25].

Following the steps outlined by Alexieva et al., [26] the TCA (0.1% w/v) technique was used to analyse the H_2_O_2_ contents. A 5 cm^3^ TCA solution was added to an ice bath containing 500 mg of leaf tissue. Following a 15-minute centrifugation at 12,000 rpm of this mixture, 0.5 mL of the supernatant was combined with 1 mL of potassium iodide and 0.5 mL of potassium phosphate buffer (100 mM) (1 M). The absorbance was read at 390 nm [27].

### Antioxidant enzymes

Each replicate’s 500 mg of leaf material was extracted in 10 mL of potassium phosphate buffer. The 50 mM potassium phosphate buffer (pH 7.8), H_2_O_2_ (40 mM), guaiacol (20 mM), and enzyme extract 0.1 mL made up the POD reaction mixture. For one minute, the absorbance change at 470 nm was measured after each 20 s interval. An absorbance change of 0.01 in one minute equalled one unit of POD [28].

To test SOD activity, 20 mL of the sample was mixed with 50 M NBT (nitroblue tetrazolium chloride), 1.3 M riboflavin, 13 mM methionine, 75 M EDTA, and 50 mM phosphate buffer. Test tubes containing this solution were exposed to light at a rate of 78 mol m2 s1 for 15 min before readings at 560 nm were collected [29].

### Ascorbic acid (AsA) content

The Mukherjee and Choudhury [30] approach was used to measure the AsA content of leaves. 0.6 mL of 6% TCA solution was used to homogenize 0.25 g of fresh leaf material. Next, the homogenate underwent a 20 min centrifugation at 10,000 g. 2 mL of an acidic dinitrophenyl hydrazine solution (2% concentration) were added to 4 mL of leaf extract. The combination also included a drop of 10% thiourea produced in 70% ethanol. The prepared mixture was cooked for 20 min at 95 °C in a water bath. A UV visible spectrophotometer was used to read the Abs of the final coloured material at 530 nm after cooling the mixture and reacting it with 5 mL of H_2_SO_4_ (80%).

### Analysis of tocopherol contents

A modified approach based on the Bakers and Myers [31] method was employed to quantify the tocopherol content in the leaves. Fresh leaf samples (0.5 g) were thoroughly mixed with a 2:1.6 (v/v) solution of petroleum ether and ethanol (10 mL). The mixture was subsequently centrifuged at 10,000 x g for 20 min. Next, 1 mL of the resulting solution was combined with 200 L of 2dipyridyl in ethanol (2%) and thoroughly mixed. The mixture was then left in the dark for five minutes. Subsequently, 4 mL of distilled and deionized water was added to the mixture and carefully stirred. The spectrophotometric measurement was conducted at 520 nm. By utilizing the tocopherol content, a standard curve was generated, enabling the calculation of the tocopherol content.

### Growth attributes

Observations were recorded on shoot and root lengths using a measuring tape, as well as the fresh and dry weights of roots and shoots of three replicates from each treatment. Plants were dried to a consistent weight in an oven set at 68 °C for a week to determine dry weight [11].

### Yield profile

To determine the various yield parameters, two plants per replication were harvested at maturity. When cobs reached physiologic maturity, they were removed from the plants and placed in sunshine. Manual labour was used to extract the grains from the cobs. The yield characteristics were estimated, including the 100kernel weight, cob length, number of rows per cob, and overall number of kernels per cob [11].

### Statistical analysis

The data was inputted into a Microsoft Excel sheet using Costat version 6.3 for conducting two-way analysis of variance research, developed by Cohort Software in Berkeley, CA, USA. The Spearman correlation matrix and principal component graphs were generated using XLSTAT version 2014, an add-in by Addinsoft based in Paris, France [32].

## Results

### Effect of n-Fe_2_O_3_seed priming on the water relations of maize plants

**Fig. 1:**
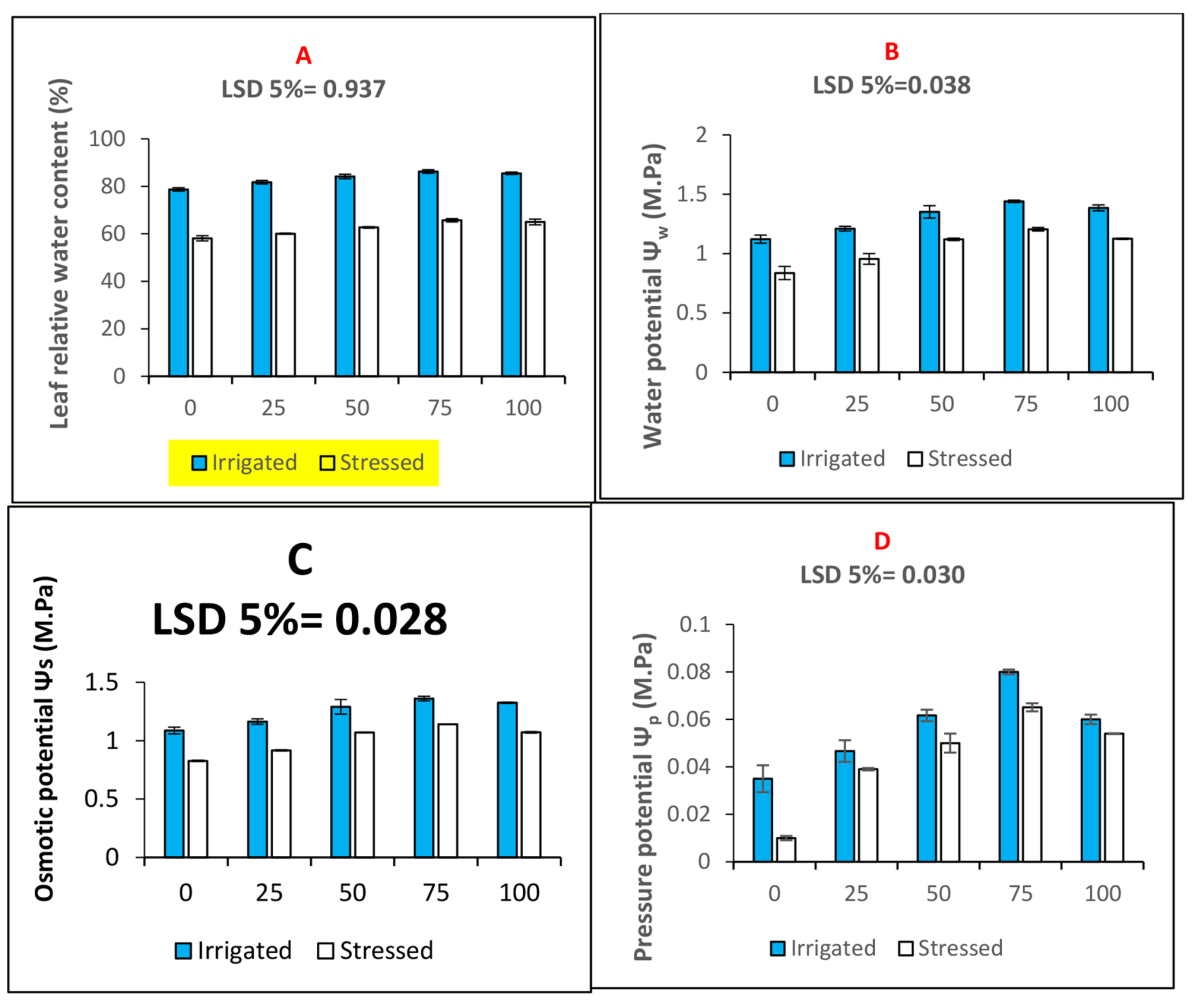
Bar charts (mean ± S.E; *n* = 3) showing leaf relative water content and water relations of maize plants subjected to seed priming with n-Fe_2_O_3_ under stressed and irrigated conditions. (**A**) Leaf relative water content percentage (**B**) Water potential (**C**) Osmotic potential and (**D**) Pressure potential. The x-axis denotes the priming treatment concentrations of n-Fe_2_O_3_ in mg. L^− 1^: 0, 25, 50, 75, and 100

The leaf relative water contents were decreased significantly under water deficit conditions (Fig. 1A). Seed priming with n-Fe_2_O_3_ increased leaf relative water contents. Furthermore, the water potential (Fig. 1B), osmotic potential (Fig. 1C) and pressure potential (Fig. 1D) of the maize plants were improved significantly under drought stress due to seed priming treatments (Table 2). Seed priming with 75 mg. L^− 1^ proved the best concentration in improving leaf water relations (Fig. 1).

**Table 2:** Statistical analysis through two-way analysis of variance results presenting information on mean square and *p* values of different variables of maize plants raised from n-Fe_2_O_3_ primed seeds

Variation Source	^a^df	LRWC	Ψ_w_	Ψ_s_	Ψ_w−_Ψ_s_	A	E	A/E	g_s_
Water Stress (WS)	1	3294.232^b^***(0.000)	0.481***(0.000)	2.143***(0.000)	0.593***(0.000)	210.998***(0.000)	2.633***(0.000)	205.188***(0.00)	262454.633***(0.000)
Priming Treatment (PT)	4	59.248***(0.000)	0.118*** (0.000)	0.011***(0.000)	0.058*** (0.000)	53.883*** (0.000)	0.026*** (0.000)	153.461***(0.000)	1312.089*** (0.000)
WS X PT Interaction	4	0.486 ns (0.537)	0.00069 ns (0.620)	0.035***(0.000)	0.043***(0.000)	0.790**(0.02)	0.032*** (0.000)	1.024*** (0.000)	496.123*** (0.000)
Error	20	0.606	0.001	0.0005	0.043	0.126	0.003	0.142	13.255
Variation Source	^***a***^ ***df***	**SDW**	**RDW**	**RFW**	**SFW**	**RL**	**SL**	**T.Chl.**	***A/*** **g**_**S**_
Water Stress (WS)	1	0.047 ns (0.238)	2.581* (0.030)	1022.188***(0.00)	13.342***(0.000)	0.086*** (0.000)	0.091*** (0.000)	770.5*** (0.000)	0.085***(0.000)
Priming Treatment (PT)	4	1.757*** (0.000)	7.734*** (0.000)	229.461***(0.000)	0.240*** (0.000)	0.715**(0.001)	0.875***(0.000)	60.326***(0.000)	0.0004*** (0.000)
WS X PT	4	0.065 ns(0.127)	0.311 ns(0.645)	1.999 ns (0.7028)	0.129ns (0.299)	168.166***(0.232)	0.095***(0.000)	11.166***(0.000)	0.0002*** (0.000)
Error	20	0.032	0.492	3.651	0.099	110	0.003	1.598	0.00002
Variation Source	*df*	**TF**	**Toc**	**CL**	**NKRPC**	**TNOKC**	**100 KW**		
Water Stress (WS)	1	87.462***(0.000)	6705.008***(0.000)	21.889 ns (0.080)	10.812 ns (0.636)	144,907*** (0.000)	110.592*** (0.000)		
Priming Treatment (PT)	4	0.719*** (0.000)	34.688***(0.000)	113.947*** (0.000)	9.866* (0.024)	67,861*** (0.000)	20.571*** (0.000)		
WS X PT	4	1.382*** (0.000)	15.262***(0.000)	3.929 ns(0.652)	0.8 ns (0.883)	1574.112**(0.739)	6.442**(0.005)		
Error	20	0.036	1.425	6.332	2.8	3178	1.274		

^a^df= degrees of freedom; LRWC = Leaf relative water content; Ψ_w_ = water potential; Ψ_s_ = solute potential; Ψ_w_-Ψ_s_ = pressure potential; *A* = number of molecules of CO_2_ assimilated; *E* = number of water molecules of water lost through transpiration; A/E = Leaf water use efficiency; g_s_= Stomatal conductance; A/g_s_= Leaf intrinsic water use efficiency; SDW = shoot dry weight, RFW = root fresh weight; SFW = soot fresh weight; RL = root length; SL = shoot length; T.Chl = Total chlorophyll; TA = total anthocyanin; MDA = malondialdehyde contents; AsA = Ascorbic acid contents; H_2_O_2_ = hydrogen peroxide values; SOD = superoxide dismutase; TF = Total flavonoids; Toc = Tocopherols; CL = cob length; NKRPC = number of grain (Kernel) rows per cob; TNOKC = total number of kernels per cob; 100KW = 100 kernel weight; POD = peroxidase; RDW = root dry weight; SCs = sugar contents

^b^ *, ** and ***=significant at 0.05, 0.01, and 0.001 levels, respectively

### Effect of n-Fe_2_O_3_ seed priming on gas exchange parameters of maize plants

**Fig. 2:**
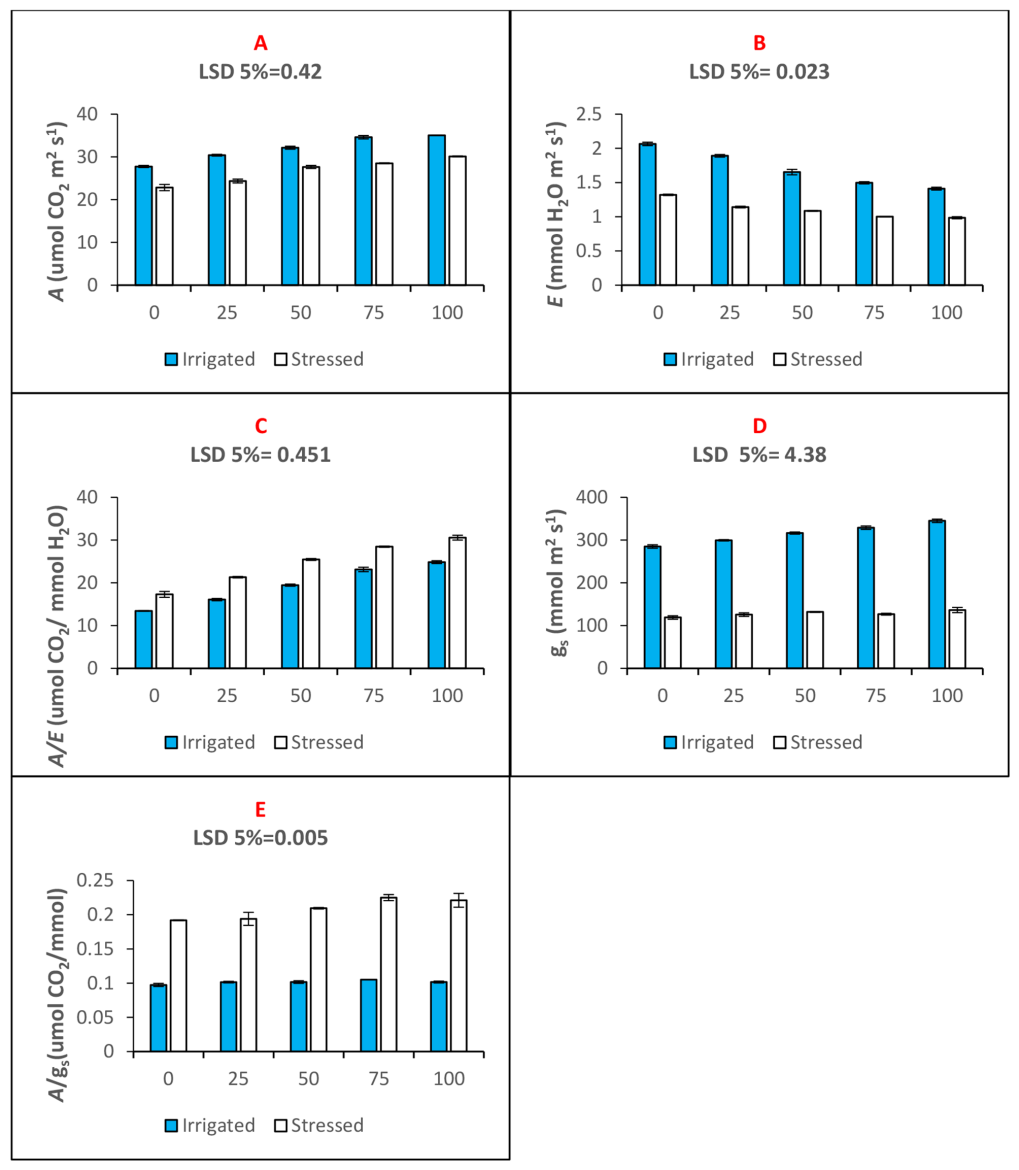
Bar charts (mean ± S.E; *n* = 3) showinggas exchange parameters of maize plants raised through seed priming use of n-Fe_2_O_3_ under drought and water. (**A**) *A* = leaf net photosynthetic rate; (**B**) *E* = leaf transpiration rate; (**C**) *A/E* = leaf water use efficiency; (**D**) g_s_ = leaf stomatal conductance; (**E**) A/g_s_ = leaf intrinsic water use efficiency.The x-axis denotes the priming treatment concentrations of n-Fe_2_O_3_ in mg. L^− 1^: 0, 25, 50, 75, and 100

Gas exchange parameters of maize plants was recorded in terms of net CO_2_ assimilation (Fig. 2A), transpiration rate (Fig. 2B), and stomatal conductance (Fig. 2C). The parameters were used to study leaf water use efficiency (Fig. 2D) and stomatal conductance (Fig. 2E). Under drought stress CO_2_ assimilation decreases however seed priming with n-Fe_2_O_3_ increases CO_2_ assimilation and decreases transpiration of water. The increase in CO_2_ assimilation and simultaneous decrease in transpiration indicates better physiological water use efficiency (A/E) of maize plants under drought stress (Fig. 2). Similarly, better stomatal conductance was induced indicating stress tolerating response induced in the maize plants as a results of seed priming treatments that also improved the leaf intrinsic water use efficiency (A/gs). Improvement in water use efficiency was treatments specific. We observed that seed priming with 75 mg. L^− 1^ concentration of n-Fe_2_O_3_ is optimum in inducing stress tolerance response (Fig. 2; Table 2).

### Effect of n-Fe_2_O_3_ seed priming on total chlorophyll, flavonoids, soluble sugars and anthocyanins contents

**Fig. 3:**
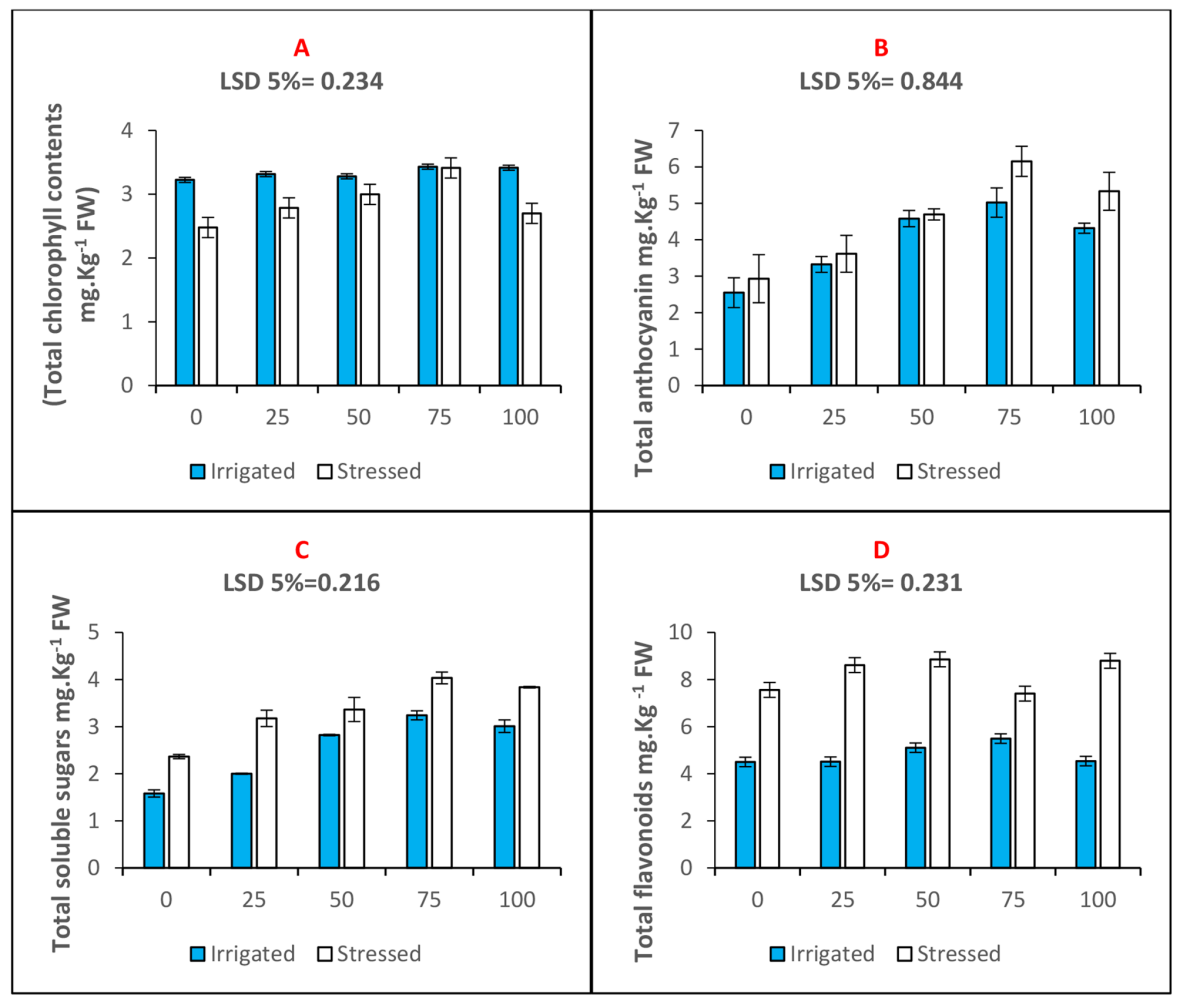
Bar charts (mean ± S.E; *n* = 3) showing pigments and biochemical parameters of maize plants raised through seed priming use of n-Fe_2_O_3_ under drought and water (**A**) Total chlorophyll (**B**)Total anthocyanin (**C**) Total soluble sugars (**D**)Total flavonoids. The x-axis denotes the priming treatment concentrations of n-Fe_2_O_3_ in mg. L^− 1^: 0, 25, 50, 75, and 100

In the present study, total chlorophyll and total anthocyanin contents of maize plants raised through n-Fe_2_O_3_ primed seeds were evaluated. The total chlorophyll content was decreased by 23% in maize plants upon the imposition of drought stress (Fig. 3A). Seed priming with 75 mg. L^− 1^n-Fe_2_O_3_ improved total chlorophyll content by 37% under drought stress compared to a 0 mg. L^− 1^ control (Table 2). The contents of total anthocyanin (Fig. 3B) and total soluble sugars (Fig. 3C) were found to be elevated under drought stress by 13 and 33%, respectively. Seed priming with n-Fe_2_O_3_ further improved the content of these parameters. The 75 mg. L^− 1^seed priming treatments increased the total anthocyanin and total soluble sugar contents by 45% and 38%, respectively. Furthermore, seed priming with 75 mg. L^− 1^n-Fe_2_O_3_ increased the content of total flavonoids in maize plants by 24% and 22% under well-irrigated and water deficit environments (Fig. 3D; Table 2).

### Effect of n-Fe_2_O_3_ seed priming on osmotic stress markers and antioxidant defence system of maize plants

**Fig. 4:**
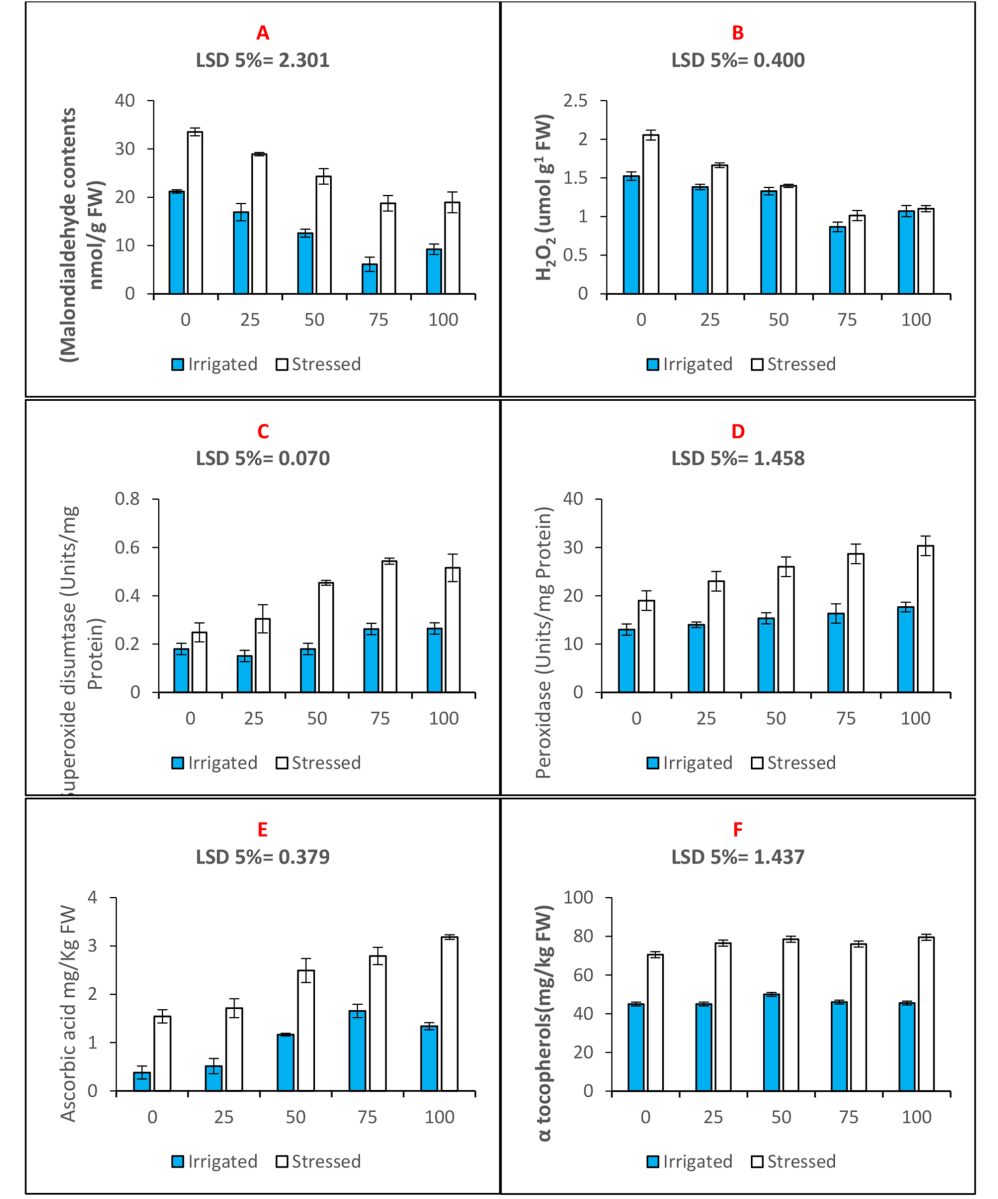
Bar charts (mean ± S.E; *n* = 3) showing osmotic stress markers: (**A**) malondialdehyde contents, (**B**) hydrogen peroxide contents, enzymatic antioxidants: (**C**) superoxide dismutase, (**D**) peroxidase activities, and antioxidant vitamins: (**E**) Ascorbic acid and (**F**) Tocopherols contents in maize plants raised with n-Fe_2_O_3_ under irrigated and stressed conditions. The x-axis denotes the priming treatment concentrations of n-Fe_2_O_3_ in mg/L: 0, 25, 50, 75, and 100

Drought increased the contents of hydrogen peroxide (by 34.8%) and malondialdehyde (by 58%) as quantified in the leaves of maize plants (Fig. 4). Seed priming with n-Fe_2_O_3_ at 75 mg. L^− 1^ concentration decreased the values of hydrogen peroxide and lipid peroxidation product malondialdehyde by 64% and 44%, respectively, under drought stress. Theactivities of SOD (Fig. 4C) and POD (Fig. 4D) were found to be elevated under drought stress by 27% and 31%, respectively. Seed priming with all trial concentrations further increased the functioning of antioxidant defence enzymes; however, the 100 mg. L^− 1^concentration caused the maximum increase in the activities of these antioxidant enzymes. Under drought stress, the activities of SOD and POD further increased by 68% and 37%, respectively (Fig. 4; Table 2).

Similarly, shoot vitamin status in terms of ascorbic acid and tocopherol content was increased by 75% and 36%, respectively, under drought stress. Seed priming with a 75 mg. L^− 1^ concentration of n-Fe_2_O_3_ improved the ascorbic acid contents by 65% and 36% under well-irrigated and water deficit conditions, respectively (Fig. 4E). The contents of alpha tocopherol were improved by 7% and 2% in the maize plants, respectively, through 75 mg. L^− 1^ priming treatment under watered and water-stressed environments (Fig. 4F; Table 2).

### Effect of n-Fe_2_O_3_ seed priming on growth variables of the maize plants

**Fig. 5:**
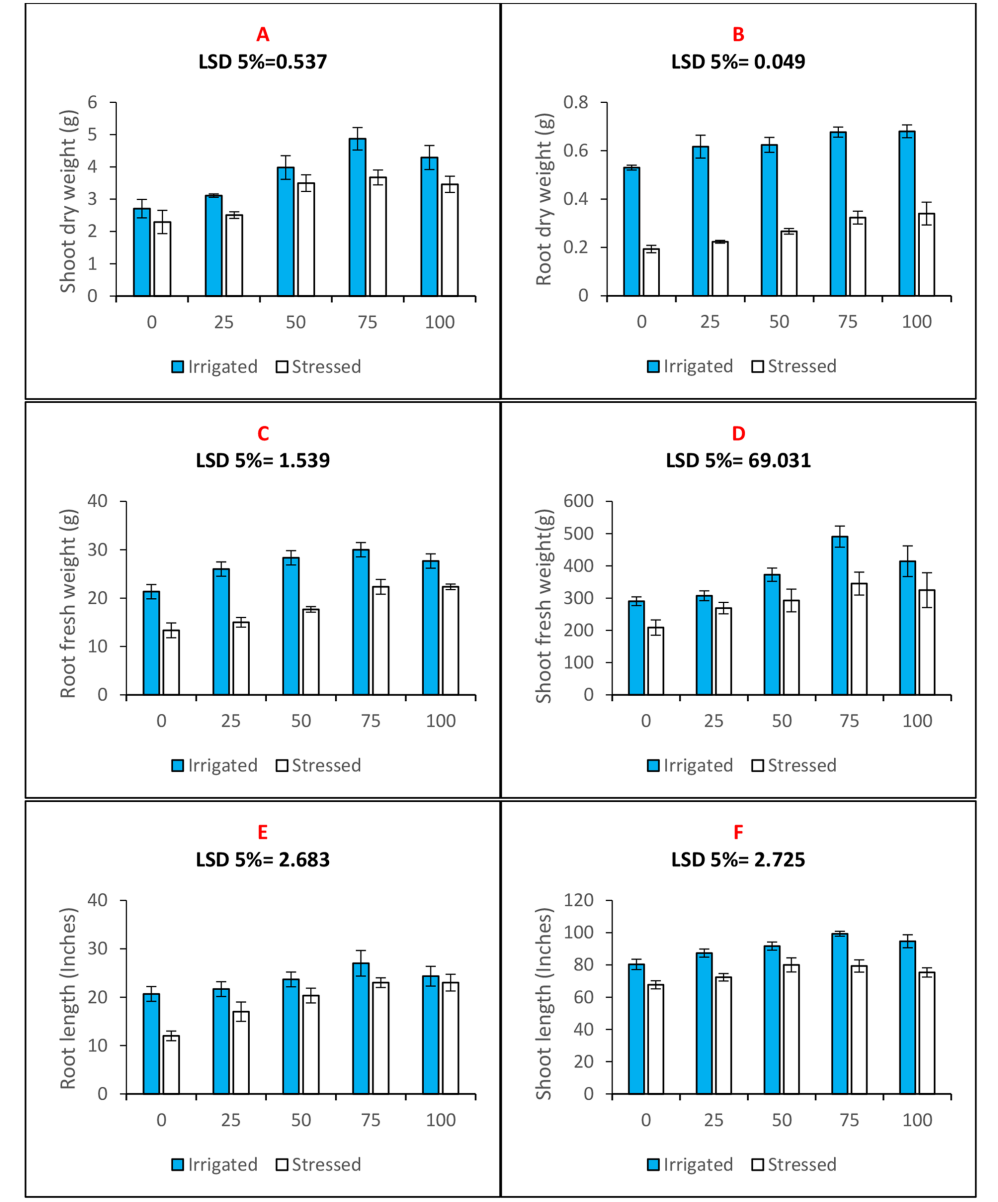
Bar charts (mean ± S.E; *n* = 3) showing growth attributes of maize plants raised through seed priming use of n-Fe_2_O_3_ under drought and water (**A**) shoot dry weight, (**B**) root dry weight (**C**) root fresh weight, (**D**) shoot fresh weight (**E**) root length and (**F**) shoot length in maize plants raised with n-Fe_2_O_3_ under irrigated and stressed conditions. The x-axis denotes the priming treatment concentrations of n-Fe_2_O_3_ in mg/L: 0, 25, 50, 75, and 100

Drought stress caused a reduction in shoot (Fig. 5A) and root dry weights (Fig. 5B) were found to decrease under the influence of drought stress by 15% and 63%, respectively. Similarly, drought reduced root fresh weight (Fig. 5C) and shoot fresh weight (Fig. 5D) by 37.5% and 28%, respectively. Seed priming with n-Fe_2_O_3_ significantly affected the root and shoot biomass of the maize plants (Table 2). All the treatment levels differentially improved the biomass of maize plants; however, seed priming with n-Fe_2_O_3_ at a concentration of 75 mg. L^− 1^ proved the best treatment in raising the biomass. Seed priming with 75 mg. L^− 1^n-Fe_2_O_3_ concentration increased shoot fresh weight and root fresh weight by 69% and 40%, respectively, under watered conditions. Seed priming with 75 mg. L^− 1^n-Fe_2_O_3_ concentration increased shoot dry weight and root dry weight by 69% and 27%, respectively, under well irrigated conditions. Seed priming with 75 mg. L^− 1^n-Fe_2_O_3_ concentration increased shoot fresh weight and root fresh weight by 65% and 67%, respectively, under water deficit conditions. Seed priming with 75 mg. L^− 1^n-Fe_2_O_3_ concentration increased shoot dry weight and root dry weight by 60% and 67%, respectively, under water deficit conditions. Plant height in terms of root length (Fig. 5E) and shoot length (Fig. 5F) were monitored and found to decrease by 41% and 15% under drought stress. All the seed priming treatments significantly raised the root length and shoot length seed priming with n-Fe_2_O_3_in both water stressed and well irrigated plots (Table 2).

### Effect of n-Fe_2_O_3_ priming on agronomic features of maize plants

**Fig. 6:**
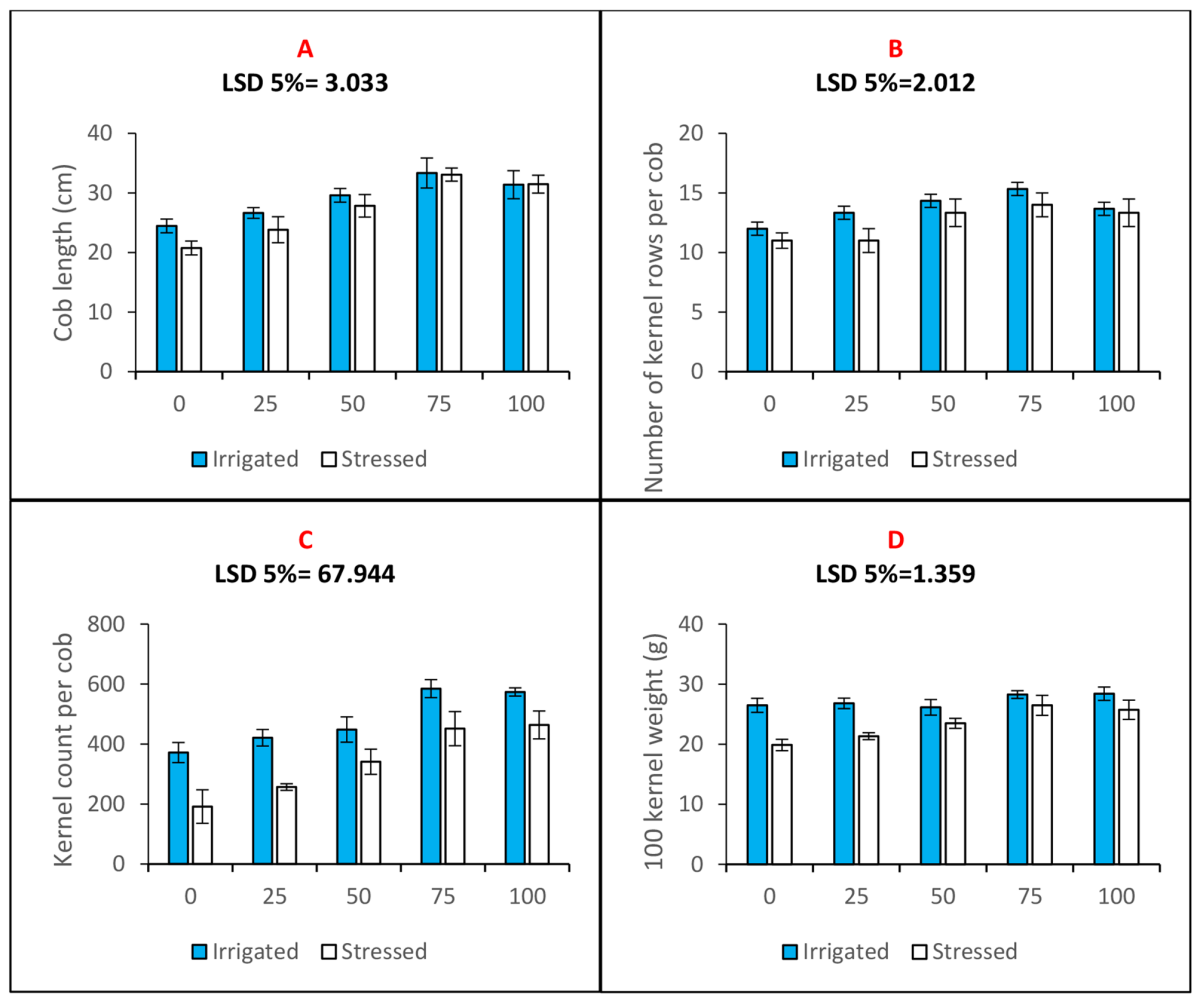
Bar charts (mean ± S.E; *n* = 3) showing studied agronomic features of maize plants (**A**) Cob length (**B**) Number of kernels rows per cob (**C**) Kernel count per cob and (**D**) 100 Kernel weight in maize plants raised with n-Fe_2_O_3_ under irrigated and stressed conditions. The x-axis denotes the priming treatment concentrations of n-Fe_2_O_3_ in mg/L: 0, 25, 50, 75, and 100

Cob length (Fig. 6A), the number of rows of kernels on each cob (Fig. 6B), the number of kernels on each cob (Fig. 6C), and the weight of 100 kernels (Fig. 6D) were all recorded as yield attributes. Data as in Fig. 6, illustrates how yield characteristics of maize plants cultivated in water-deficit conditions drastically dropped. Cob length, kernel rows per cob, kernels per cob, and 100kernel weight all decreased as a result of drought stress by 15%, 9%, 48%, and 24%, respectively. n-Fe_2_O_3_ primed seeds produced maize plants that had a better yield profile in terms of the characteristics that were measured. The 75 mg. L^− 1^ priming concentration of n-Fe_2_O_3_ improved the cob length, number of kernel rows per cob, number of kernels per cob, and 100kernel weight under well-irrigated conditions by 36%, 27%, 57%, and 17%, respectively. The 75 mg. L^− 1^ priming concentration of n-Fe_2_O_3_improved the cob length, number of kernel rows per cob, number of kernels per cob, and 100kernel weight by 59%, 27%, 87%, and 33%, respectively, under water deficiency circumstances (Fig. 6).

**Fig. 7:**
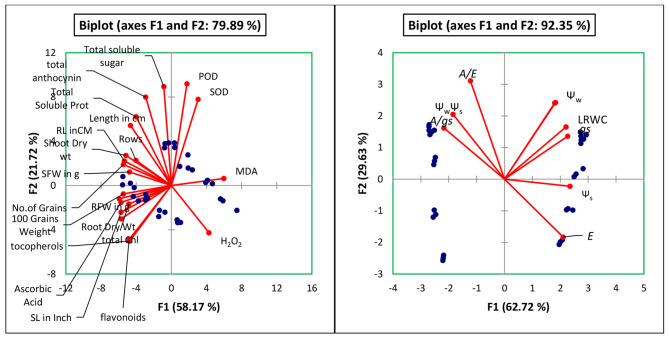
Principal component analysis loading charts of various parameters studied of maize plants raised through seed priming with NFe2O3 under drought and water. LRWC = Leaf relative water content; Ψ_w_ = water potential; Ψ_s_ = solute potential; Ψ_w−_Ψ_s_ = pressure potential; *A* = number of molecules of CO_2_ assimilated; *E* = number of water molecules of water lost through transpiration; A/E = Leaf water use efficiency; g_s_= Stomatal conductance; A/g_s_= Leaf intrinsic water use efficiency

Principal component analysis has been shown in Fig. 7. The analysis predicts a clear percentage of the two principal factors in defining the variance. Significant results have been shown in the table on several occasions by the seed priming treatments. Spearman correlation matrix among the variables has been presented in the Tables 3 and 4.

**Table 3:** Spearman correlation matrix for the variables and growth and yield parameters of maize plants grown under water stress and well irrigated subplots from iron oxide nanoparticles primed seeds

Variables	Cob Length	Kernel rows	No. of Grains	100 Grains Weight	Shoot Dry weight	Root Dry weight	Root fresh weights	Shoot fresh weight	RL (cm)	SL (Inch)
Cob Length (cm)	1									
Kernel rows	0.678*	1								
No. of Grains	0.766*	0.581*	1							
100 Grains Weight	0.682*	0.478*	0.812*	1						
Shoot Dry weight	0.711*	0.651*	0.746*	0.592*	1					
Root Dry weight	0.507*	0.487*	0.814*	0.849*	0.685*	1				
RFW in g	0.654*	0.567*	0.782*	0.847*	0.758*	0.914*	1			
SFW in g	0.564*	0.487*	0.692*	0.592*	0.776*	0.706*	0.705*	1		
RL (cm)	0.778*	0.559*	0.785*	0.773*	0.686*	0.734*	0.848*	0.606*	1	
SL (inches)	0.571*	0.520*	0.769*	0.797*	0.729*	0.938*	0.883*	0.730*	0.714*	1
total Chlorophyll	0.492*	0.382*	0.569*	0.759*	0.544*	0.710*	0.741*	0.523*	0.674*	0.717*
total anthocyanin	0.814*	0.526*	0.525*	0.362*	0.552*	0.186	0.352	0.365*	0.538*	0.262
Total soluble sugar	0.457*	0.113	0.303	0.021	0.383*	-0.101	0.039	0.247	0.328	-0.064
Malondialdehyde	-0.675*	-0.532*	-0.844*	-0.855*	-0.765*	-0.932*	-0.957*	-0.784*	-0.867*	-0.910*
Ascorbic Acid	0.588*	0.522*	0.781*	0.849*	0.718*	0.883*	0.899*	0.618*	0.762*	0.857*
Hydrogen peroxide	-0.690*	-0.470*	-0.692*	-0.579*	-0.669*	-0.513*	-0.615*	-0.498*	-0.650*	-0.468*
Superoxide dismutase	0.072	-0.181	-0.313	-0.474*	-0.250	-0.694*	-0.560*	-0.228	-0.217	-0.640*
Peroxidase	0.257	-0.039	-0.062	-0.299	-0.002	-0.515*	-0.385*	-0.104	-0.041	-0.486*
Total flavonoids	0.324	0.368*	0.545*	0.667*	0.499*	0.826*	0.783*	0.501*	0.549*	0.828*
tocopherols	0.283	0.414*	0.548*	0.636*	0.527*	0.835*	0.774*	0.513*	0.503*	0.832*

Values with a * are significantly correlated at alpha 0.05%

**Table 4:** Spearman correlation matrix for the variables and biochemical parameters of maize plants grown under water stress and well irrigated subplots from iron oxide nanoparticles primed seeds

Variables	Total chlorophyll	total anthocyanin	Total soluble sugar	Malondialdehyde	Ascorbic Acid	Hydrogen peroxide	Superoxide dismutase	Peroxidase	Total flavonoids	tocopherols
total Chlorophyll	1	0.244	-0.012	-0.737*	0.754*	-0.470*	-0.470*	-0.394*	0.723*	0.600*
total anthocyanin	0.2440	1	0.690	-0.366*	0.287	-0.640*	0.363*	0.531*	0.039	-0.006
Total soluble sugar	-0.0127	0.690*	1	-0.126	0.027	-0.434*	0.588*	0.794*	-0.310	-0.309
Malondialdehyde	-0.737*	-0.366*	-0.126	1	-0.900*	0.615*	0.515*	0.315	-0.744*	-0.737*
Ascorbic Acid	0.754*	0.287	0.027	-0.900*	1	-0.615*	-0.551*	-0.376*	0.802*	0.809*
Hydrogen peroxide	-0.470*	-0.640*	-0.434*	0.615*	-0.615*	1	0.079	-0.222	-0.351	-0.328
Superoxide dismutase	-0.470*	0.363*	0.588*	0.515*	-0.551*	0.0798	1	0.835*	-0.672*	-0.726*
Peroxidase	-0.394*	0.531*	0.794*	0.315	-0.376*	-0.2220	0.835*	1	-0.650*	-0.622*
Total flavonoids	0.723*	0.039	-0.310	-0.744*	0.802*	-0.3515	-0.672*	-0.650*	1	0.918*
tocopherols	0.600*	-0.006	-0.309	-0.737*	0.809*	-0.3282	-0.726*	-0.622*	0.918*	1

Values with a * are significantly correlated at alpha 0.05%

## Discussion

### Plant water relations

Seed priming with n-Fe_2_O_3_ improves leaf water relations and water use efficiency. This is due to the role of n-Fe_2_O_3_ in the expression of aquaporin genes, forming aquaporin channels in the root cells, facilitating the water uptake through the roots [33, 34]. n-Fe_2_O_3_also alter the lipid composition of the membranes, changing their fluidity. The change in fluidity and composition of the membrane promotes energetic forces which drive water into the cell, improving plant water relations in terms of leaf relative water content and water potential. Priming causes osmotic adjustment through changes in membrane fluidity, influencing the potential of endo-membranous tissues by compensating for drought-induced loss in water potential and leaf relative water content [35].

### Gas exchange parameters

Seed priming with n-Fe_2_O_3_improves the net photosynthetic rate and carbon dioxide assimilation [36]. This is due to iron involved in the synthesis of ribulose1, 5-bisphosphate, which is an important enzyme in carbon dioxide fixation. It can be assumed that seed priming with n-Fe_2_O_3_increased biosynthesis of ribulose1, 5-bisphosphate, which resulted in higher assimilation of carbon dioxide. Iron is essentially a part of chlorophyll biosynthesis and it is an absolute requirement for the proper functioning of photosystems. Thus, iron deficiency results in the declined performance of electron transport chain carriers. Supplying iron through controlled release by seed priming might be a valuable choice to boost carbon dioxide assimilation and gas exchange parameters. Stomatal conductance was improved in maize plants raised through n-Fe_2_O_3_primed seeds [37]. Better stomatal conductance manifests improved stomatal opening. It has been reported that iron deficiency leads to poor absorption of cations such as K^+^. In plants, potassium ions are crucial to the regulation of the opening and closing of stomata. Furthermore, the accumulation of osmolytes such as total soluble sugars and better starch metabolism due to iron oxide nanoparticle treatment might have resulted in better stomatal conductance and lowering transpiration induced loss of water [38].

### Impact on biochemical attributes, vitamins, metabolites, and osmolytes accumulation

In plants, several important metabolic processes, such as DNA synthesis, energy production, and conversion, are dependent on the availability of iron. Seed priming with n-Fe_2_O_3_ promotes Ca^2+^signalling in plants. In plants, Ca^2+^acts as a secondary messenger and, thereby, it brings about changes in transcriptional reprogramming, leading to improved secondary metabolism [39]. Improved metabolism contributes to more production of flavonoids, glucosinolates, and phenolic. Furthermore, seed priming improves starch metabolism, resulting in an accumulation of soluble sugars that act as osmolytes in maize plants, inducing drought tolerance. Seed nanopriming with n-Fe_2_O_3_ brings about the optimisation of ROS levels. The optimum levels of ROS improve the secondary metabolism in plants. The optimisation of ROS levels promotes the activation of other signalling molecules such as jasmonic acid, which brings about their effect on secondary metabolism of plants [40]. That leads to more accumulation of plant secondary metabolites such as flavonoids, as reported in our results. Increased production of n-Fe_2_O_3_ mediated by flavonoids leads to the production and accumulation of anthocyanin. In the present research, we noted an increased content of anthocyanin under drought stress, which was further enhanced upon seed priming with n-Fe_2_O_3_. In plants, anthocyanins are drought stress induced ROS scavengers. The anthocyanin prevents the build-up of ROS, which leads to efficient water homeostasis under drought stress, enabling plants to tolerate drought stress [41]. Under drought stress conditions, plants undergo various physiological and biochemical changes to adapt and survive. One of these changes involves the accumulation of antioxidants such as tocopherols (vitamin E) and ascorbic acid (vitamin C) to counteract the damaging effects of reactive oxygen species (ROS) that accumulate in plant tissues. Furthermore, iron oxide seed priming has been shown to increase the uptake of essential nutrients such as iron, which is required for the synthesis of important biomolecules including tocopherols and ascorbic acid. Therefore, it is possible that iron oxide seed priming can increase the production of tocopherols and ascorbic acid in plants grown under drought stress conditions. Overall, the mechanism behind how iron oxide seed priming might increase tocopherols and ascorbic acid contents under drought stress involves both increased antioxidant enzyme activity and improved nutrient uptake [42].

### Efficacy in enhancing total chlorophyll contents and biomass

The use of n-Fe_2_O_3_ in seed priming improved the chlorophyll content of maize plants significantly in the current study. Iron acts as important structural atoms in the synthesis of d-aminolevulinic acid, which acts as precursor in the biosynthesis of chlorophyll pigment in plants [4]. Due to its role in the production of d-aminolevulinic acid, iron indirectly contributes to higher levels of total chlorophyll. Although iron is among the most abundant metals in the environment, it is the third most limiting metal in plant nutrition. Improved plant biomass was observed upon seed priming treatment in the form of n-Fe_2_O_3_. The presence of more photosynthetic pigments and enhanced root proliferation may have contributed to the rise in plant biomass [42]. Iron acts as a cofactor for cytochrome and certain oxygenase and thus boosts endogenous metabolism of maize plants [43]. Thus, the enhanced metabolism plays a role in raising a plant’s biomass. Furthermore, the iron nutrition in plants results in mobilization of soil nutrient and due to enhanced water availability, the soil nutrients acquisition is improved due to courtesy of transpiration pull [44]. Priming resulted in enhanced accumulation of ascorbic acid which is an important antioxidant molecule. Similarly, tocopherols are proven antioxidant candidates against oxygen toxicities. From the enhanced contents of these vitamins upon seed priming with n-Fe_2_O_3_, it can be assumed that maize plants are now better in mitigating the water stress [45].

### Efficacy of n-Fe_2_O_3_ in depressing stress indicators and boosting antioxidant defence

The antioxidant enzymes SOD and POD were further stimulated by seed priming with n-Fe_2_O_3_. These results are consistent with an experiment conducted by Das et al., [45] in which rice seeds were primed with nano iron pyrite. Both superoxide dismutase and peroxidase have iron in their fundamental structures. SOD defends cells against abiotic stress by converting superoxide radicals (a kind of ROS) into molecular oxygen, whereas POD scavenges hydrogen peroxide by turning it into water [46]. When exposed to an iron deficit, the antioxidant enzymes’ activity diminishes [47]. Thus, from improved activities of these antioxidant enzymes, it can be assumed that n-Fe_2_O_3_seed priming might have initiated a stress tolerance response in maize plants, which was quite evident as findings of the present research report decreased contents of hydrogen peroxide and by product of lipid peroxidation.

### Impact of n-Fe_2_O_3_seed priming use on yield of maize

The yield and production of maize in terms of kernel parameters were evaluated. Seed priming with n-Fe_2_O_3_mitigated the adverse impacts of drought on maize yield. The improved maize yield is due to a stress relieving response mediated by n-Fe_2_O_3_, which includes improved water relations, gas exchange parameters, increased chlorophyll contents, improved antioxidant enzymes, and accumulated osmolytes. Water deficit environments reduce the activity of enzymes such as oxygenase, carboxylase, and rubisco activase, leading to poor carbon dioxide fixation [48, 49]. This ultimately leads to poor agronomic performance due to pollen sterility, ovary abortion, and impaired grain outcomes. However, seed priming with n-Fe_2_O_3_ improves water availability and boosts the activities of these enzymes, resulting in improved photosynthesis and hence better yields [50]. These findings are consistent with earlier research by Yasmeen et al., [51], who found that seed priming with copper and iron particles significantly improved wheat spike length, grain count per spike, and endosperm content. Additionally, an important factor in improving yield quality is the increase in plant photosynthetic capacity brought about by n-Fe_2_O_3_. Because iron boosts metabolism by increasing enzymatic activity and because priming treatments affect the expression of many genes during flowering and fruiting, seed priming with n-Fe_2_O_3_ is an effective strategy under compromised yields [52].

## Conclusion

In the present research, we proved the hypothesis that priming the maize seeds with n-Fe_2_O_3_might increase the growth, production, and water use efficiency of plants grown on land experiencing drought. Shoot vitamin status of maize plants was improved and osmolytes such as total soluble sugars and total anthocyanin accumulated under water stress, inducing tolerance to drought in the experimental maize plants raised through seed priming with n-Fe_2_O_3_. Seed nanopriming resulted in depressing the content of hydrogen peroxide and malondialdehyde and enhancing the production of chlorophyll. Future climates will be drier and agricultural lands will be under intense pressure to increase production. In such fragile lands, seed priming with bio rational and cost-effective nanomaterials such as n-Fe_2_O_3_might be a sustainable strategy compared to conventional fertilizers, which are also a source of nutrient pollution. Using a pro-fertilizer might reduce nutrient pollution since the fertilizers applied on croplands leach in the surrounding soil and water causing eutrophication and nutrient pollution. Under such circumstances seed priming can be eco-friendly practice.

### Electronic supplementary material

Below is the link to the electronic supplementary material.

Supplementary Material 1

## Data Availability

All data generated or analysed during this study are included in this published article.
