# Supplementary material for: Optimizing water relations, gas exchange parameters, biochemical attributes and yield of water-stressed maize plants through seed priming with iron oxide nanoparticles
Source: BMC Plant Biol. 2024 Jun 29;24:624. doi: 10.1186/s12870-024-05324-w (PMC11218355; doi:10.1186/s12870-024-05324-w)
Supplement: Supplementary file 1 — Supplementary Material 1 [file 12870_2024_5324_MOESM1_ESM.docx]

**Table S1. Seed priming treatments**

| Treatment | Concentration of Iron oxide nano powder mg/L |
| --- | --- |
| Control | One Littre Distilled Water (D.W) |
| 25 ppm | 25 mg of Nano powder / L of D.W |
| 50 ppm | 25 mg of Nano powder / L of D.W |
| 75 ppm | 25 mg of Nano powder / L of D.W |
| 100 ppm | 25 mg of Nano powder / L of D.W |
